# Prediction of response to pegylated-interferon-α and ribavirin therapy in Chinese patients infected with different hepatitis C virus genotype

**DOI:** 10.1186/1743-422X-9-123

**Published:** 2012-06-19

**Authors:** Xiaoyan Guo, Zhixin Zhao, Junqiang Xie, Qingxian Cai, Xiaohong Zhang, Liang Peng, Zhiliang Gao

**Affiliations:** 1 Department of Infectious Diseases, The Third Affiliated Hospital of Sun Yat-Sen University, 600 Tian He Road, Guangzhou, 510630, P.R. China; 2 Key Laboratory of Tropical Disease Control, Ministry of Education, Sun Yat-sen University, Guangzhou, Guangdong, 510080, Peoples’ Republic of China

**Keywords:** IL-28B, Hepatitis C, Relapse, Sustained virological response, Treatment

## Abstract

**Background:**

The standard treatment for patients with chronic hepatitis C (CHC), pegylated interferon-α (PEG-IFN) plus ribavirin (RBV) does not provide a sustained virological response (SVR) in all patients. Genetic variations at the interleukin 28B (IL-28B) locus are important in predicting outcome following therapy in CHC patients.

**Results:**

We investigated the role of IL28B variations (rs8099917) in response to PEG-IFN-α/RBV treatment and evaluated its association with the risk of the null virological response (NVR) and relapse (REL) in different viral genotypes. We found that the overall distributions of the genotype among the SVR, NVR, and REL groups were significantly different (P<0.001). Patients with the TG genotype had an increased risk of NVR and REL (OR=6.45 95% CI =2.88–14.47, P<0.001 for NVR; OR=2.51, 95% CI =1.29–4.86, P=0.006 for REL, respectively), and patients with the GG genotype had a further increased risk of NVR and REL (OR=12.04, 95% CI =3.21–45.13, P<0.001 for NVR; ,OR=4.30, 95% CI =1.21–15.13, P=0.017 for REL, respectively). G variant genotypes (TG+GG) also had an increased risk of NVR and REL, and there was a significant trend for a dose-effect of G allele on the risk of NVR and REL (P<0.05). The SVR rate in TT higher than in TG+GG was more pronounced in those patients infected with non-G1 compared to the patients infected with G1. The treatment response did differ based on the rs8099917 genotype in patients with different viral genotypes, compared with patients infected with the non-G1, the G1 infected patients had an increased risk of NVR and REL (OR=2.03 95% CI =1.03–4.01, P=0.04 for NVR and OR=2.58, 95% CI =1.35–4.94, P=0.004 for REL, respectively). Moreover, multivariate regression analysis show that the rs8099917 G allele was the only independent factor significantly associated with a NVR and REL.

**Conclusion:**

This study suggests that host genetic polymorphisms rs8099917 in the vicinity of IL-28B is the most important predictor of treatment response of PEG-IFN-α/RBV for HCV patients in China.

## Background

Hepatitis C is a global health problem which affects a significant proportion of the world, accounting for 3% of the total population [1,2]. There are approximately 40 million people in China infected with the hepatitis C virus (HCV), accounting for 1.7% of the Chinese population. Presently, the standard treatment for patients with chronic hepatitis C (CHC) is pegylated interferon-α (PEG-IFN- α) and ribavirin (RBV) [3,4]. In the United States and Europe, 42%–52% of patients with HCV genotype 1 achieve a sustained virological response (SVR) [5]. However, the response rate was much higher in China when treated with the corresponding regimen [6,7]. It has long been suspected that host genetic factors are key determinants for the treatment of CHC.

Recently, some genome-wide association studies (GWAS) have identified that the single nucleotide polymorphism (SNP) rs8099917 effectively predicts responses to IFN therapy [8-11]. Importantly, those studies used similar genotyping techniques and analytical methods and included different ethnic groups, the outcome was definite. The SNP is located on chromosome 19q13, which is 3 kilobases downstream of the interleukin-28B (IL-28B) gene that encodes type III interferon (IFN-λ3)[12,13]. The IL-28B genotype frequency reportedly varies according to ethnicity and is associated with viral responses to PEG-IFN-α/RBV therapy against HCV genotype 1 (G1)-infected patients from North America, Europe, and Japan [14-16]. No systematic explanation exists for the viral genotype-specific differences which exist in response to treatment. The importance of theses IL28B variants for Chinese patients and for the non-1 viral genotype (non-G1) remains unknown. If non-G1 viral genotypes have a proclivity to infect patients with a specific IL28B genotype, it would be useful to predict an individual’s response either before treatment or during the early stages of treatment. Some patients have withdrawn from IFN-based therapy because of undesirable side effects [17]. Prediction of the probable effectiveness of IFN therapy therefore plays an essential role in a clinical setting. Adverse drug reactions can be avoided in patients unlikely to benefit from treatment, and the substantial cost of PEG-IFN-α/RBV treatment could be reduced [11]. The aim of this study was to determine the influence of the rs8099917 variations in the response of PEG-IFN-α/RBV among HCV genotypes in a southern China population.

## Results

### Baseline characteristics

The clinical characteristics and the treatment responses of the 236 infected with HCV included in this study are presented in Table 1. Overall, there was no significant difference in our study cohort among the SVR, NVR, and REL respect to age, gender, BMI, liver enzyme levels, infection time and HCV RNA viral load(all *P* > 0.05). However, the HCV genotype frequencies were significantly different among the SVR, NVR, and REL groups (*P* = 0.006). Of the CHC patients, 53%(126/236) were infected with HCV viral G1, 47%(110/236) were infected with HCV viral non-G1 (13% with genotype 2, 4% with genotype 3, and 30% with genotype 6,respectively). The proportion of the G1 genotype in NVR and REL groups was higher than in SVR group (61.7%, 67.2%, and 44.3%, respectively). Moreover, severe fibro sis was significantly associated with NVR or REL (*P* = 0.007).

**Table 1:** Demographic and biochemical features of patients

**Variable**	**SVR**	**NVR**	**Rel**	***P*value**
**Total no. of subjects**	131	47	58	
**Age (years)**	44.96 ± 10.85	40.87 ± 14.18	44.76 ± 10.97	0.106
**Gender: male (%)**	79(60.3%)	36(76.6%)	35(60.3%)	0.116
**BMI**	22.17 ± 2.94	22.01 ± 2.64	22.77 ± 3.43	0.352
**ALT(U/L)**	60(33–119)	57(38–89)	59(27–95)	0.315
**AST(U/L)**	49(34–120)	49(35–69)	45(28–73)	0.219
**Platelet count (×10**^**4**^**/mm**^**3**^**)**	17.48 ± 5.42	17.16 ± 6.40	18.42 ± 11.35	0.641
**HCV genotype 1 (%)**	58(44.3%)	29(61.7%)	39(67.2%)	0.006
**Fibrosis**^**a**^**: F3-F4 (%)**	16(29.1%)	16(66.7%)	10 (45.5%)	0.007
**Infection time**^**b**^**(year)**	5(2–13)	5(3–12)	6(3–11)	0.952
**Virus load (log IU/ml)**	6.18 ± 1.17	6.08 ± 0.82	6.12 ± 1.05	0.617

a: 101 patients had ultrasound-guided percutaneous liver biopsies, all biopsy specimens were used for the histologic diagnosis b:duration of exposure on risk factors to initial anti-viral therapy.

### The distribution and association of IL28B genotype with response to PEG-IFN-α/RBV treatment

The genotype and allele distributions of the IL28B rs8099917 polymorphisms are summarized in Table 2. The overall distributions of the genotype among the SVR, NVR, and REL groups were significantly different (*P* < 0.001). The frequencies of TT was significantly higher in the patients achieved SVR than in those achieved NVR and REL (65.6% in SVR, 21.3% in NVR, 41.4% in REL, respectively). Logistic regression analysis showed that compared with patients with the TT genotype, TG genotype had an increased risk of NVR and REL (odds ratio [OR] = 6.45 95% confidence interval [CI] =2.88–14.47, P < 0.001 for NVR; OR = 2.51, 95% CI =1.29–4.86, P = 0.006 for REL, respectively), GG genotype had a further increased risk of NVR and REL (OR = 12.04 95% CI =3.21–45.13, P < 0.001 for NVR; OR = OR = 4.30, 95% CI =1.21–15.13, P = 0.017 for REL, respectively). Complicated TG with GG genotype together, G variant genotypes (TG + GG) also had an increased risk of NVR and REL, and there was a significant trend for a dose-effect of G allele on the risk of NVR and REL. Moreover, compared with T allele carriers, G allele carriers had a significantly higher risk of NVR and REL (OR = 3.73, 95% CI =2.24–6.21, *P* < 0.001 for NVR; OR = 2.23, 95% CI =1.37–3.45, *P* = 0.001 for REL, respectively).

**Table 2:** The distribution and association of IL28B genotype with response to PEG-IFN-α/RBV treatment

	**SVR**	**NVR**	**Rel**	**NVR vs. SVR**	***P*value**	**Rel vs. SVR**	***P*value**
	**N,%**	**N,%**	**N,%**	**OR(95%CI)**		**OR(95%CI)**	
SNP genotype							
TT	86(65.6%)	10(21.3%)	24(41.4%)	1.0 (Reference)		1.0 (Reference)	
TG	40(30.5%)	30(63.8%)	28(48.3%)	6.45(2.88–14.47)	<0.001	2.51(1.29–4.86)	0.006
GG	5(3.8%)	7(14.9%)	6(10.3%)	12.04(3.21–45.13)	<0.001	4.30(1.21–15.13)	0.017
TG+GG	45(34.4%)	37(78.3%)	34(58.6%)	7.07(3.22–15.52)	<0.001	2.71(1.44–5.11)	0.002
Allele							
T allele	212(80.9%)	50(53.2%)	76(65.5%)	1.0 (Reference)		1.0 (Reference)	
G allele	50(19.1%)	44(46.8%)	40(34.5%)	3.73(2.24–6.21)	<0.001	2.23(1.37–3.45)	0.001

### Treatment response according to IL28B genotype in different HCV genotypes

When the treatment response was stratified according to the viral genotype, there were more non-G1 patients with the rs8099917 TT genotype with a SVR compared to the number of patients with the rs8099917 TG + GG genotype. Compared with patients infected with G1, the SVR rate in TT higher than in TG + GG was more pronounced in those patients infected with non-G1 (77.9% in TT and 39.4% in TG + GG for non-G1 versus 60.5% in TT and 38.6% in TG + GG for G1). Moreover, the treatment response did differ based on the rs8099917 genotype in patients with different viral genotypes. Compared with patients infected with non-G1, G1 infected patients had an increased risk of NVR and REL (OR = 2.03 95% CI =1.03–4.01, *P* = 0.04 for NVR; OR = 2.58, 95% CI =1.35–4.94, *P* = 0.004 for REL, respectively) (Table 3).

**Table 3:** Treatment response according to IL28B genotype in different HCV genotypes

**Genotype**	**SVR**	**NVR**	**REL**	**P value**	**NVR vs. SVR**	**Rel vs. SVR**
	**N,%**	**N,%**	**N,%**		**OR(95%CI)**	**OR(95%CI)**
Non-G1	73(66.4%)	18(16.3%)	19(17.3%)		1.0 (Reference)	1.0 (Reference)
TT	60(77.9%)	7(9.1%)	10(13.0%)	<0.001		
TG+GG	13(39.4%)	11(33.3%)	9(27.3%)			
G1	58(46.0%)	29(23.0%)	39(31.0%)		2.03(1.03–4.01)	2.58(1.35–4.94)
TT	26(60.5%)	3(7.0%)	14(32.6%)	0.006		
TG+GG	32(38.6%)	26(31.3%)	25(30.1%)			

### Factors associated with NVR and REL

To further examine the relative contribution of factors associated with NVR and REL, we used a logistic regression model. When adjusting for HCV genotype, baseline levels of HCV RNA load and ALT, baseline age, gender, infection time, fibrosis, the G allele of rs8099917 was the most significant factor for predicting NVR and REL (OR = 14.02, 95% CI = 3.35–58.65, *P* < 0.0001 for NVR versus SVR and OR = 2.27, 95% CI = 1.13–4.58, *P* = 0.022 for REL versus SVR, respectively). This result suggests that the rs8099917 G allele may was a most important predictive factor for NVR or REL before PEG-IFN-α/RBV therapy (Table 4).

**Table 4:** Factors associated with NVR and REL by multivariate logistic regression

**Factors**	**NVR vs. SVR**			**REL vs. SVR**		
	**Odds ratio**	**95% CI**	***P*value**	**Odds ratio**	**95% CI**	***P*value**
G allele	14.02	3.35–58.65	<0.0001	2.27	1.13–4.58	0.022
Age	0.98	0.94–1.01	0.228	0.98	0.95–1.02	0.292
BMI	1.01	0.88–1.17	0.861	1.05	0.94–1.17	0.437
Gender	1.96	0.77–4.98.49	0.158	0.91	0.44–1.90	0.806
Infection time(year)	1.01	0.92–1.10	0.892	1.00	0.94–1.07	0.945
HCV genotype	0.77	0.31–1.95	0.587	1.85	0.86–3.98	0.113
Virus load	0.83	0.57–1.20	0.318	0.81	0.60–1.10	0.174
ALT	1.00	0.99–1.01	0.087	0.99	0.98–1.00	0.048
Fibrosis	3.02	0.91–10.01	0.070	1.70	0.48–6.01	0.409

## Discussion

In the present study, we showed the existence of different rs8099917 genotype frequencies and HCV viral genotypes in patients with different treatment responses. Carriers with the rs8099917 TT genotype and non-G1 HCV genotype had a high chance of achieving SVR and a lower likelihood of either NVR or REL. In contrast, carriers with the rs8099917 TG/GG genotype and G1 HCV genotype had a high risk of NVR or REL. Combining the two pretreatment predictors, IL-28B genetic variants and HCV genotype accurately predicted treatment efficacy of every patient before therapy. This strategy could conceivably play a very important role in the HCV treatment decision-making process because of the high frequency of the favorable allele and the high frequency of SVR in China [18]. This study is anticipated to open a new window for genotype-based personalized medicine for Chinese patients with CHC.

Nowadays, several studies have reported that genetic variants near IL-28B are associated with response to treatment of chronic HCV infection with a combination of PEG-IFN and RBV [8,9,11,19]. The IL-28B gene allele frequency was reported to vary according to ethnicity. In the current study, we found that G variants (TG + GG) carriers had a significantly higher risk of NVR and REL. Moreover, logistic regression analysis showed that G allele was an independent predictive factors of NVR and REL, which was similar to reports from Japanese patients in previous studies [11,20]. These results may provide useful information for a personalized treatment regimen.

In the current study, it is noteworthy that a high rate of Chinese patients with the TT genotype of rs8099917 attained a SVR. Similarly, Tanaka Y, *et al.* reported that rs8099917 had a strong association with response to therapy and the TT genotype of the rs8099917 polymorphism was associated with a high SVR rate [11]. Suppiah *et al.* also reported that the highest rate of SVR was in the group of patients with the TT genotype of rs8099917 and that the presence of the G allele was the most powerful predictive factor for NVR [9] . Overall, our results were in agreement with the results reported by Suppiah *et al.* and Tanaka *et al.* Logistic regression model identified the rs8099917 G allele as the most significant factor for predicting NVR (OR = 14.02), whereas the prediction of REL using the SNP had lower statistical power (OR = 2.27) compared to NVR prediction, indicating that these SNPs are strongly associated with the outcome of NVR. The REL patients with the TT genotype might be able to achieve a SVR following prolonged therapy with PEG-IFN/RBV. Clinically, many factors, such as age, gender, pretreatment HCV RNA levels, higher pretreatment AST levels, liver fibrosis status, and insulin resistance are important factors in the outcome of PEG-IFN-α/RBV therapy [5,21-23]. Nevertheless, this result was not observed in our study, it is possible that such a finding is attributable to relatively small numbers of subjects in the cohort.

It is widely accepted that both host and virus factors can influence the SVR[7,24]. Ge *et al.* reported that among Caucasian patients with G1, the rs12979860 (3 kilobases upstream of the IL-28B gene) wild CC genotype was an independent predictor favoring SVR [10]. McCarthy *et al.* demonstrated a similar finding with respect to off-treatment viral loads in Caucasian patients with G1 [25]. Our study included 47% non-G1 infected patients, and surprisingly, the percent of patients with a favorable IL-28B genotype was higher in the non-G1 group (77.9%). There is the possibility that the rs8099917 TT genotype may help select patients with non-G1 infections. The influence of host genotype could be slightly stronger among individuals infected by the non-G1 than among those infected by the G1. We found that G1 infected patients had an increased risk of NVR and REL than non-G1, and this data generated from the current study could provide important information for patients who either would or would not benefit from PEG-IFN-α/RBV treatment before starting treatment. However, these results need additional confirmation in other cohorts.

It is anticipated that this study, which identified a highly genetic association between NVR and REL in patients with different viral genotypes, will have a lasting impact, particularly in the development of individualized treatment strategies. Also, the higher frequency of the TT genotype in Chinese patients may explain (at least partially) why Chinese patients have superior responses to PEG-IFN-α/RBV therapy than other nationalities. The strong associations between IL-28B (rs8099917) genotypes and SVR are consistent in different ethnic groups [10]. The IL-28B genotype may guide clinical decision making regarding whether or not to initiate therapy, how long to continue therapy for avoiding unpleasant side effects associated with treatment. Nevertheless, there are also NVR (9.1%) and REL (13.0%) patients, even when the favorable TT genotype and non-G1 was identified in patients included in the current study. These results indicate that further prospective studies comparing the treatment efficacy are warranted.

The major limitation of this study is the small number of included cases resulting in low statistical power. Further, whether or not geographic variations in the HCV core protein mutations in the interferon sensitivity-determining region contribute to a relatively much higher SVR rate in the current study remains uncertain [23,26,27]. In this study it was not possible to compare HCV genotype frequencies and IL-28B genotypes between individuals with either cleared or chronic infection because samples from the acute phase of HCV infection were unavailable in individuals with spontaneous HCV clearance.

## Conclusion

In conclusion, host genetic polymorphisms rs8099917 in the vicinity of IL-28B is the most important predictor of treatment response of PEG-IFN-α/RBV for Chinese HCV patients. Furthermore, viral genotype testing prior to HCV therapy could provide important information towards the development of an individualized HCV treatment regimen.

## Methods

### Patients

The study protocol was approved by the institutional review boards of Sun Yat-Sen University. Written informed consent was obtained from each participant after a full explanation of the study.

We retrospectively recruited 236 patients from Chinese with CHC and different HCV genotype infection who underwent the current standard-of-care regimens between 2006 and 2010 in the outpatient clinic of the Third Affiliated Hospital of Sun Yat-Sen University. The diagnoses of CHC were based on previously described criteria [28]. The patients were positive for anti-HCV antibody and serum HCV RNA for > 6 months. All eligibale subjects were treated with PEG-IFN-α-2a at a fixed dose of 180 μg/week and ribavirin 800–1,200 mg/day (i.e., 800 mg for patients <65 kg; 1,000 mg for patients weighing 65 to 85 kg; 1,200 mg for patients weighing 85 to 105 kg). Patients with genotype 1 and 6 were treated for 48 weeks and patients with genotype 2 or 3 were treated for 24 weeks. Quantitative HCV RNA(Cobas Amplicor Hepatitis C Virus Test, V.2.0; Roche Diagnostics, Branchburg, New Jersey, USA; detection limit: 50 IU/mL) and alanine aminotransferase(ALT) levels were evaluated 4, 12, 48, and 72 weeks after the start of anti-viral therapy. An autoimmune panel and thyroid function tests were performed every 3 months during treatment. After completing the therapy, the patients were followed- up for 24 weeks and the therapeutic effectiveness was evaluated. Patients who withdraw from therapy due to undesirable side effects were excluded. Patients included in the SVR group had normal ALT levels and no evidence of viremia 24 weeks after completion of IFN therapy). Those included in the NVR group had HCV RNA levels detectable during the completed period of the treatment, and patients in the REL group had HCV RNA reappearance during the follow-up period but who had initially achieved an end-of-treatment virological response. In total, 101 patients had ultrasound-guided percutaneous liver biopsies. All biopsy specimens were used for the histologic diagnosis. Disease staging was defined according to Desmet [11,29] with ranking from F0 (absence of fibrosis) to F4 (cirrhosis stage).

### Genomic DNA isolation

Blood was collected in EDTA tubes using standard procedures. Genomic DNA was extracted from 200 μL of the cell suspension with Omega kits (Omega-Tek, Mansfield, OH, USA). All DNA preparations were stored at −70 °C until further use.

### Polymerase chain reaction (PCR) and rs8099917 SNP genotyping

The PCR bands of interest were excised from agarose gel, and the DNA fragments were purified with gel extraction kits (Omega-Tek). The purified fragments were sequenced with rs8099917 sense (5'-CCCACTTCTGGAACAAATCGTCCC-3) and rs8099917 antisense (5'-TCAACCCCACCTCAAATTATCCTA-3') sequencing primers from Shanghai Invitrogen Biotechnology Co., Ltd. (Beijing, China). The genotypes of the patients were determined using ABI TaqMan SNP genotyping assays (Applied Biosystems, Foster City, CA, USA). Allele discrimination was achieved by detecting fluorescence using System SDS software (Applied Biosystems, Foster City, CA, USA).

### Statistical analysis

Frequencies were compared between groups using either a chi-square test with the Yates correction or the Fisher exact test. Group means, presented as mean values and standard deviations, were compared using analysis of variance and the Student’s *t*-test or Mann–Whitney *U* test. Serum HCV RNA levels were expressed after logarithmic transformation of original values. To assess the relative contribution of predictors of NVR and REL, the odds ratios (ORs) and 95% confidence intervals (CIs) were calculated. Statistical analyses were performed using the SPSS 13.0 statistical package (SPSS, Inc., Chicago, IL, USA). All statistical analyses were based on two-sided hypothesis tests with a significance level at *p* <0.05.

## Abbreviations

HCV, Hepatitis C virus IL-28B: interleukin 28B; CHC, Chronic hepatitis C; PEG-INF-α, Pegylated interferon alpha; RBV, Ribavirin; SVR, Sustained virological response; NVR, Null virological response; REL, Relapse; SNP, Single nucleotide polymorphism; GWAS, Genome-wide association studies; AST, Aspirate aminotransferase; G1, Genotype 1; non-G1, Non-1 viral genotype; ALT, Alanine aminotransferase; OR, Odds ratio; CI, Confidence interval.

## Competing interests

The authors declare that no competing interests exist.

## Authors’ contributions

XG, ZZ, and ZG conceived the study, participated in its design and coordination, and managed the preparation of the manuscript. JX and QC performed the statistical analyses and analyzed the results. XZ and LP provided the clinical data. All authors read and approved the final manuscript.

## Authors’ information

All authors are affiliated with the Department of Infectious Diseases, Third Affiliated Hospital of Sun Yat-Sen University, Guangzhou, China. All authors contributed equally to this work.
